# ATG24 Represses Autophagy and Differentiation and Is Essential for Homeostasy of the Flagellar Pocket in *Trypanosoma brucei*

**DOI:** 10.1371/journal.pone.0130365

**Published:** 2015-06-19

**Authors:** Ana Brennand, Eva Rico, Daniel J. Rigden, Patrick Van Der Smissen, Pierre J. Courtoy, Paul A. M. Michels

**Affiliations:** 1 Research Unit for Tropical Diseases, de Duve Institute, and Laboratory of Biochemistry, Université catholique de Louvain, Brussels, Belgium; 2 Departamento de Bioquímica y Biología Molecular, Campus Universitario, Universidad de Alcalá, 28871 Alcalá de Henares, Madrid, Spain; 3 Institute of Integrative Biology, Biosciences Building, University of Liverpool, Liverpool, United Kingdom; 4 Cell Biology Unit, de Duve Institute, Université catholique de Louvain, Brussels, Belgium; University of Hull, UNITED KINGDOM

## Abstract

We have previously identified homologs for nearly half of the approximately 30 known yeast Atg’s in the genome database of the human sleeping sickness parasite *Trypanosoma brucei*. So far, only a few of these homologs have their role in autophagy experimentally confirmed. Among the candidates was the ortholog of Atg24 that is involved in pexophagy in yeast. In *T*. *brucei*, the peroxisome-like organelles named glycosomes harbor core metabolic processes, especially glycolysis. In the autotrophic yeast, autophagy is essential for adaptation to different nutritional environments by participating in the renewal of the peroxisome population. We hypothesized that autophagic turnover of the parasite’s glycosomes plays a role in differentiation during its life cycle, which demands adaptation to different host environments and associated dramatic changes in nutritional conditions. We therefore characterized *T*. *brucei* ATG24, the *T*. *brucei* ortholog of yeast Atg24 and mammalian SNX4, and found it to have a regulatory role in autophagy and differentiation as well as endocytic trafficking. ATG24 partially localized on endocytic membranes where it was recruited via PI3-kinase III/VPS34. ATG24 silencing severely impaired receptor-mediated endocytosis of transferrin, but not adsorptive uptake of a lectin, and caused a major enlargement of the flagellar pocket. ATG24 silencing approximately doubled the number of autophagosomes, suggesting a role in repressing autophagy, and strongly accelerated differentiation, in accordance with a role of autophagy in parasite differentiation. Overexpression of the two isoforms of *T*. *brucei* ATG8 fused to GFP slowed down differentiation, possibly by a dominant-negative effect. This was overcome by ATG24 depletion, further supporting its regulatory role.

## Introduction

Sleeping sickness, a parasitic disease severely affecting man and cattle in sub-Saharan Africa, is caused by the protist *Trypanosoma brucei* that belongs to the Kinetoplastea clade. The life cycle of *T*. *brucei* involves sequential differentiation from the long-slender (LS) into short-stumpy (SS) forms when residing in the mammalian blood (bloodstream forms, BSF), then from the SS forms into the procyclic forms (PCF) adapted to the midgut of the tsetse fly vector. Kinetoplastids' unique features include packing of the majority of glycolytic enzymes inside peroxisome-like organelles, named glycosomes [1]. This compartmentalization is essential for the survival of the LS BSF, as well as the PCF when cultured in glucose-rich conditions [2–4].

The molecular machineries allowing formation of all organelles belonging to the peroxisome family appear well conserved. In particular, biogenesis of glycosomes is similar to that of peroxisomes in different organisms, although some important differences have been reported [5]. As in peroxisomes, the enzymatic content of glycosomes is adapted to the nutritional conditions faced by the cells. *T*. *brucei* BSF freely grow in the mammalian bloodstream and feed on its highly abundant and constantly available glucose. They depend entirely on the metabolism of glucose through glycolysis for their ATP supply, with pyruvate excreted as the major end-product [6]. In sharp contrast, PCF cells need to survive in different regions of the tsetse fly’s digestive system, where glucose supply is irregular (only being available for short periods after bloodmeals), but where amino acids, especially proline, are more abundant and constantly available [7]. As a consequence, the enzymatic content of glycosomes changes when cells differentiate from the BSF to the PCF stage, whereas mitochondrial metabolism, that is largely repressed in BSF trypanosomes, becomes predominant in PCF cells. The differences in enzymatic content between these two major stages of the parasite have been recently highlighted in a proteomics study [8]. The need of *T*. *brucei* to adapt to these different environments, especially the requirement to dispose of BSF-specific glycosomes when differentiating into PCF, is highly reminiscent of yeast adaptation to changes in carbon source. This requires the expression of a new set of proteins, some to be imported into peroxisomes, as well as the removal of redundant peroxisomes by autophagy [9].

Autophagy is a well-conserved mechanism by which cytoplasmic material, soluble proteins or entire organelles, are sequestered into the cell's degradative organelle(s), namely lysosomes or the vacuole in yeast, for degradation and recycling. Autophagy occurs at a basal level as a housekeeping process to dispose of misfolded proteins as well as senescent or inadequate organelles, so as to maintain cell fitness. Autophagy can be up-regulated under widely diverse natural conditions, not only starvation or stress, but also during cell differentiation or sporulation [10].

Specific autophagic degradation of peroxisomes, known as pexophagy, has been shown to participate in the adaptation of methylotrophic yeasts from growth on (m)ethanol to glucose as source of energy (for a review see [11]). In these cells, growth on alcohol requires multiple large peroxisomes containing alcohol oxidase, which can account for most of the cell volume. When shifted to a glucose-rich environment, pexophagy is observed. The term of pexophagy is extended to the autophagy of glycosomes, which exhibit multiple similarities to peroxisomes.

AuTophaGy-related proteins (ATGs) are required for the induction of autophagy. Some ATGs are broadly distributed, such as those involved in the two ubiquitin-like complexes (centered around ATG8 and ATG12) that participate in the expansion of the phagophore membrane. Other ATGs, mainly those responsible for pathway specificity, seem to be restricted to only one species or a narrow group of species [12].

The occurrence of autophagy in *T*. *brucei* is long known, thanks to early electron microscopic studies showing an increasing number of autophagosomes in BSF cells differentiating from the LS to the SS form [13]. Following molecular characterization of autophagy in yeasts, we identified several ATG homologs in trypanosomatids [14]. We later observed that autophagy in *T*. *brucei* was increased during starvation as well as during differentiation *in vivo* and *in vitro*. Upon *in vivo* differentiation of BSF from proliferating LS to non-proliferating SS, glycosomal aldolase showed increasing co-localization with p67, a marker for lysosomes, indicating up-regulation of autophagic degradation of the organelles; when the SS forms were induced *in vitro* to further differentiate into PCF cells, co-localization further increased [15]. These results raised the hypothesis that trypanosomes adapt their metabolism to the new conditions thanks to ‘en-bloc’ replacement of ‘old’ glycosomes optimally equipped for glycolysis with a new set of glycosomes more appropriate to the completely different nutritional conditions prevailing in the fly’s midgut [16]. Production of new enzymes in the cytosol and subsequent import into the glycosomal matrix via the action of so-called peroxins (abbreviated as PEXs) can certainly provide adaptation to such changes. However, it is also likely that part of the glycosomal population, considered as “mature organelles”, is no longer able to import newly synthesized enzymes from the cytosol, as observed for mature peroxisomes of *Hansenula polymorpha*. In these yeast cells, a significant change in nutritional conditions not only induces pexophagy, but two peroxins involved in peroxisome biogenesis, Pex14 and Pex3, also participate in their degradation [17,18]. Although the mechanism by which Pex14 is involved is not yet completely understood, exposure of its N-terminal region seems required [18]. Furthermore, peroxisome degradation in the vacuole appears to be triggered by Pex3 removal from the peroxisomal membrane followed by its degradation via the ubiquitin-proteasome system [18,19].

While there is no proof that a similar mechanism signals for degradation of glycosomes in *T*. *brucei*, their disposal via autophagy is clearly upregulated during *in vivo* and *in vitro* differentiation. In BSF, the metabolic organization, biogenesis and matrix-protein import into glycosomes are essential for parasite survival and thus validated as drug targets [20]. However, little is known about how the parasite changes the metabolic content of its glycosome population from one stage to the other. Here, we characterize the putative ATG24 in *T*. *brucei*. Its yeast ortholog (in *Pichia pastoris* and *Saccharomyces cerevisiae*) is involved in specific types of autophagy [21,22]. The same *T*. *brucei* protein was previously identified as a VPS5 by Koumandou and colleagues [23] and shown to be associated to retromer. We demonstrate in this paper that silencing of this ATG24/VPS5 homolog increases autophagosome numbers and promotes differentiation. In *T*. *brucei*, ATG24 silencing increases autophagosome numbers and promotes differentiation. Taken together, these observations demonstrate the link between the two processes and suggest that, in normal conditions, ATG24 is a repressor of autophagy and a lock of differentiation. Like *S*. *cerevisiae* Atg24 and the corresponding mammalian protein (SNX4—sorting nexin 4), *T*. *brucei* ATG24 is recruited to intracellular membranes by the phosphatidylinositol 3-kinase (PI3 kinase) activity of VPS34 and is essential for receptor-mediated endocytosis. *T*. *brucei* ATG24 is also involved in the homeostasy of the flagellar pocket. Altogether, these results thus indicate that TbATG24 may act as a link between autophagy and endocytic membrane trafficking.

## Results

### Bioinformatics analysis of *T*. *brucei* ATG24

**Fig 1:**
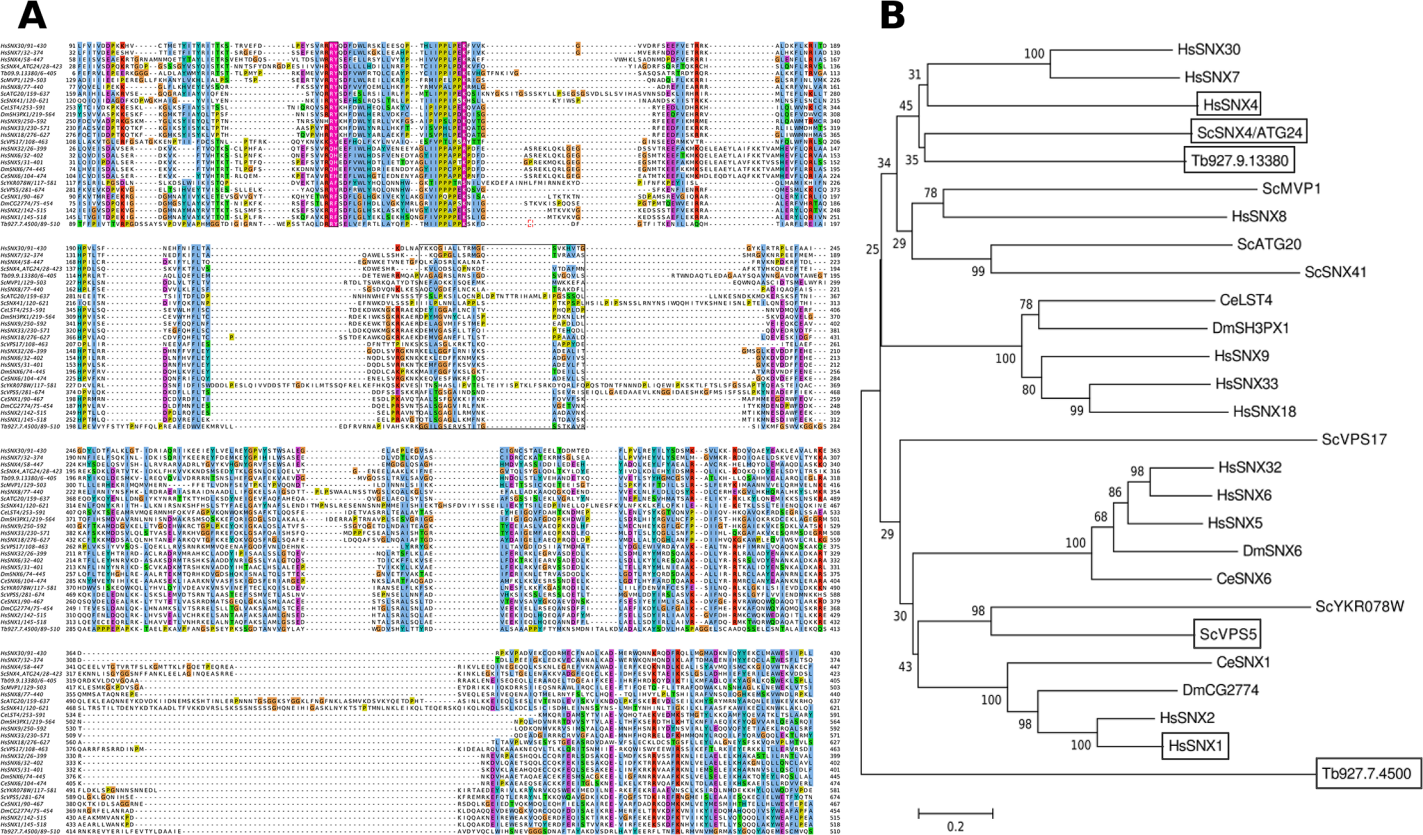
Identification of TbATG24 by sequence homology. **A**) Amino acid sequence alignment of several SNXs from different species where the domain boundaries are boxed and key PI3P-binding residues are shown. Sc—*Saccharomyces cerevisiae*, Hs–*Homo sapiens*, Ce–*Caenorhabditis elegans*, Dm–*Drosophila melanogaster* **B**) Phylogenetic tree of the two PX-BAR-containing proteins from *T*. *brucei* (Tb; one of which Tb927.9.13380, is here referred to as TbATG24) and PX-BAR containing proteins from other organisms. The relationships were inferred by the Minimum Evolution method. The branches are drawn to scale, with lengths in the same units (the number of base differences per site; indicated by the bar) as those of the evolutionary distances used to infer the phylogenetic tree. The evolutionary distances were computed as described in the Materials and Methods. The tree is bootstrapped; the numbers near the nodes indicate the percentage of replicate trees in which the associated taxa clustered together in the bootstrap test (500 replicates). Phylogenetic reconstructions using the Neighbor-Joining and Maximum Parsimony methods (S1 Fig) gave essentially the same topology. Proteins considered important for the discussion are boxed.

TbATG24 (Tb927.9.13380, formerly known as Tb09.211.4240) was identified in the databases by homology searches with *S*. *cerevisiae* Atg24 as described previously [14]. The protein shares 24% sequence identity with its yeast counterpart. However, the protein was recently identified as the *T*. *brucei* equivalent of Vps5/SNX1 [23]. To explore the relationship of TbATG24 with yeast homologs more thoroughly, sequences were aligned (Fig 1A) and three phylogenetic trees, one of which is shown in Fig 1B (the other two in the Supporting Information, S1 Fig), were constructed based on calculations with different methods. Although many bootstrapping values are low, these suggest that the *T*. *brucei* protein is evolutionarily more closely related to Atg24/SNX4 than to Vps5/SNX1.

### ATG24 is an endocytic protein

**Fig 2:**
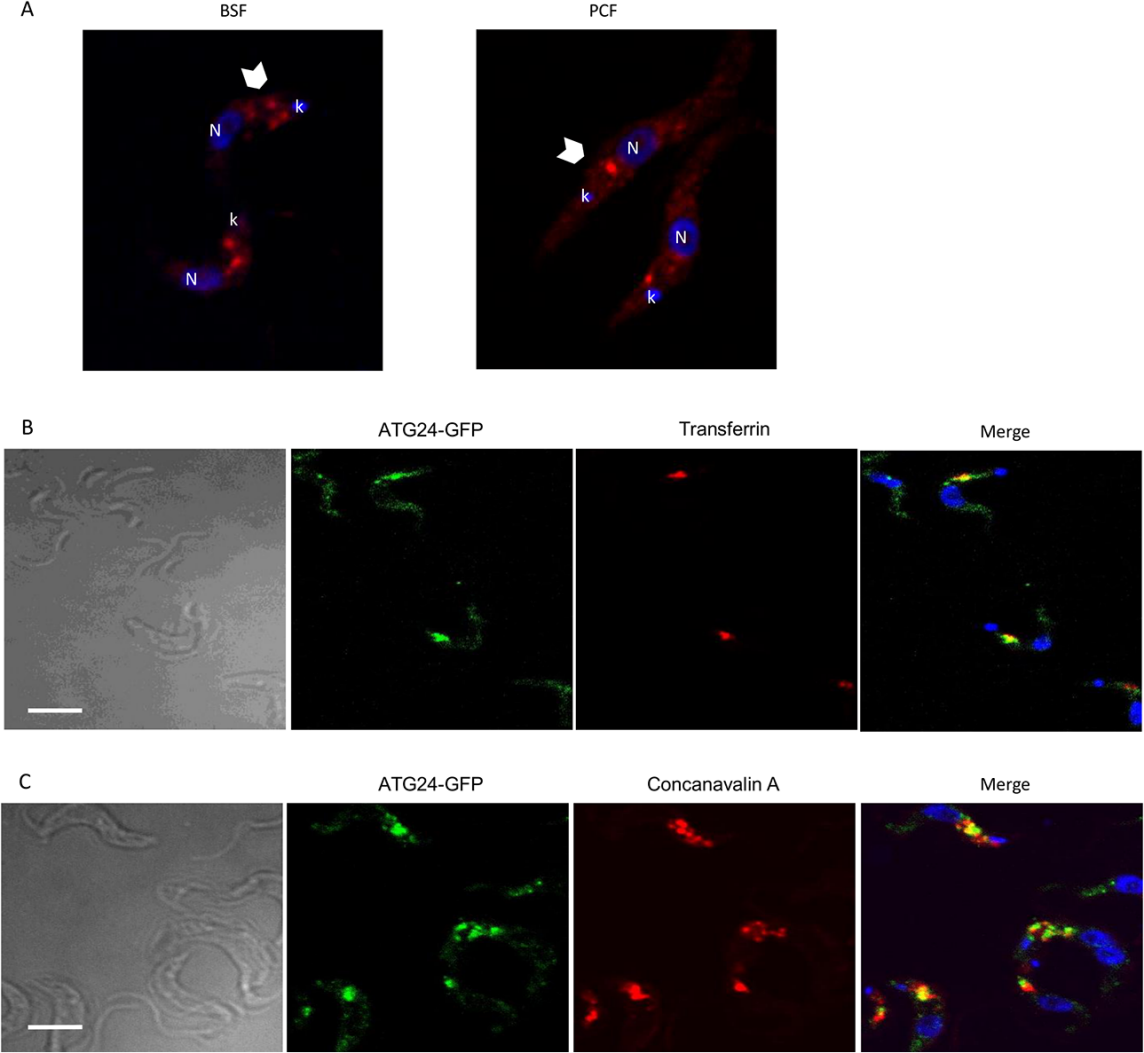
Dual localization of ATG24 in *T*. *brucei* cytosol and endosomes. **A**) ATG24 subcellular localization by immunofluorescence. Notice that ATG24 immunolabeling (in red) produces a combined dotty pattern (arrowheads) with diffuse cytosolic signal, both in BSF (left panel) and PCF wild-type cells (right panel). Blue is DAPI, N = nucleus, k = kinetoplast. **B**) Relation with endosomes in wild-type BSF cells expressing ATG24-GFP (green) incubated with the receptor-mediated endocytic tracer, Alexa568-transferrin (red), at 37°C for 30 min. Yellow in the merge at right indicates extensive colocalization. DAPI is shown in blue. **C**) Relation with endosomes by triple imaging with concanavalin A and DAPI. Wild-type BSF cells expressing ATG24-GFP (green) were incubated with the adsorptive endocytosis tracer, Alexa594-concanavalin A (red) at 37°C for 30 min. All scale bars: 5 μm. In two independent experiments the mean Pearson's correlation of colocalization observed between ATG24 and transferrin was 0.24 and with concanavalin A was 0.21. As positive and negative controls, respectively, endosomal RAB11 had a Pearson's coefficient of 0.34 and DAPI 0.13.

Rabbit antiserum, raised for this study against recombinant full-length ATG24, recognized a single band at the expected size of 48 kDa in whole-cell lysates of both LS-BSF and PCF *T*. *brucei* cells (S2A Fig). Confocal immunofluorescence (Fig 2A) as well as direct imaging of the Green Fluorescent Protein (GFP)-tagged version of the protein (Fig 2B and 2C) revealed a dual localization: diffuse in the cytoplasm (i.e. cytosolic) and concentrated in a few puncta generally clustered between the nucleus and kinetoplast, i.e. the typical position of the endocytic apparatus in *T*. *brucei*. The expression of the ATG24 with a GFP tag did not affect the growth rate of the cells (not shown). To test for association with endocytic organelles, BSF cells expressing GFP-ATG24 were incubated for up to 30 min at 37°C with fluorescent transferrin (Fig 2B) or concanavalin A (Fig 2C) as respective tracers of receptor-mediated (transferrin:receptor complex) and bulk adsorptive endocytosis (concanavalin A is a general lectin of mannose that binds surface glycoproteins). Both tracers showed partial colocalization with ATG24, as concluded from the determined Pearson correlation coefficient (see legend Fig 2), thus confirming (at least partial) endocytic localization of the protein.

### ATG24 requires VPS34 for its correct subcellular localisation

**Fig 3:**
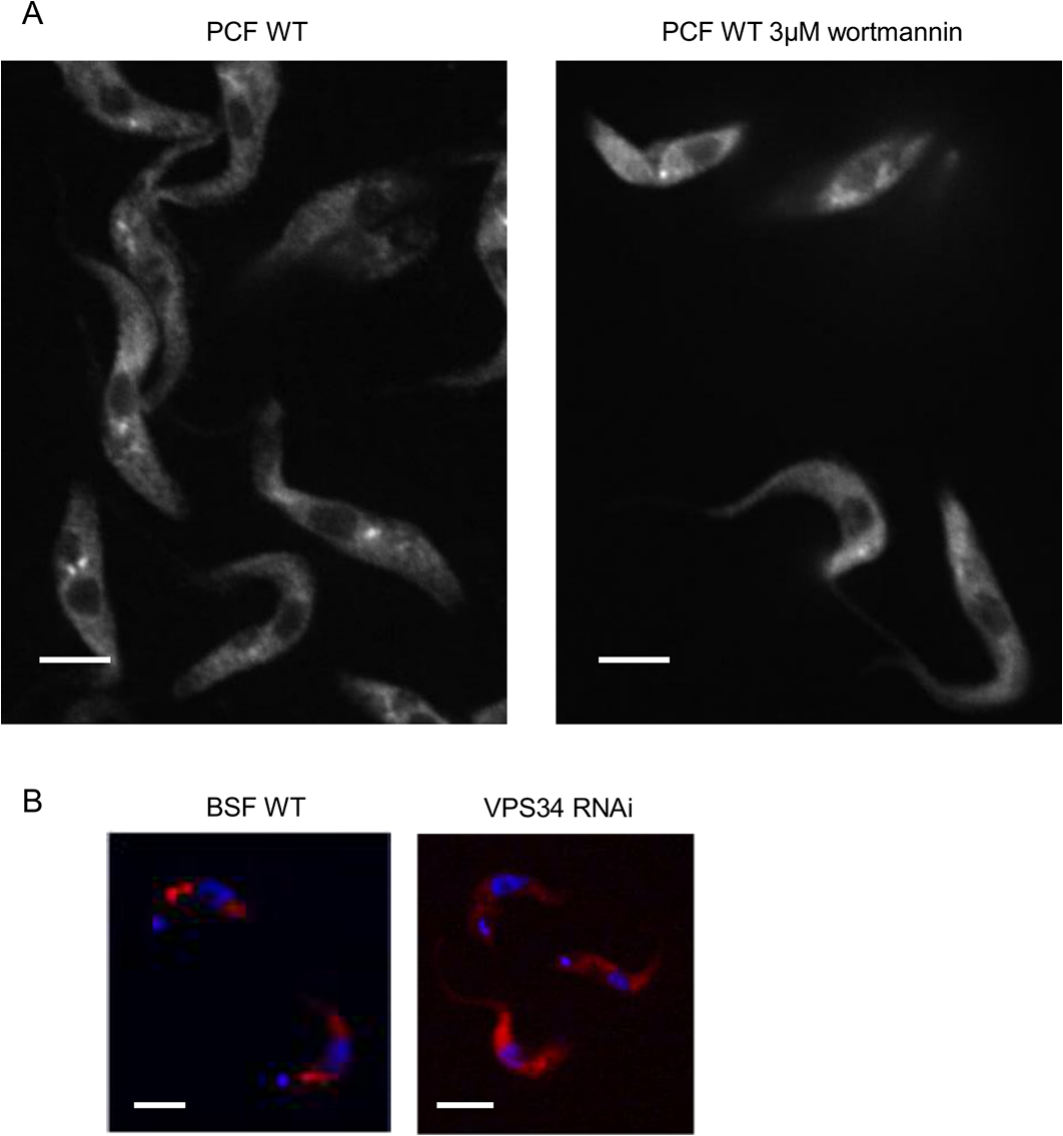
ATG24 membrane recruitment in *T*. *brucei* depends on VPS34. **A**) Pharmacological inhibition. ATG24 immunofluorescence was determined in wild-type PCF cells in the absence of wortmannin and after 1 h of incubation with 0.3 to 30 μM of this compound. Shown here are (in black and white for better contrast and resolution) non-treated trypanosomes (in the left panel) and trypanosomes treated with 3 μM wortmannin for 1 h (in the panel at the right). Quantification of the effect of wortmannin was performed in two experiments with 60 and 150 cells, respectively. **B**) VPS34 silencing. ATG24 immunofluorescence (red) in wild-type (WT) and VPS34 RNAi BSF at 24 h after induction. Blue shows DAPI. The intensity value assigned to ATG24 fluorescence in endosomes was 3.4 times higher in WT BSF than in VPS34 RNAi BSF 24-h induced cells. Results are from one representative experiment out of two where 45 WT and 10 VPS34 RNAi cells and 18 WT and 21 VPS34 RNAi cells were counted, respectively. All scale bars: 5 μm.

ATG24 contains no transmembrane region, but its N-terminal PX domain allows for its recruitment onto membranes, as shown by studies with yeast and mammalian mutant cells [21,22,24]. This domain interacts primarily with phosphatidylinositol 3-phosphate (PI3P); it also allows lower affinity binding to PtdIns(3,5)P2, whereas mammalian Snx4 also interacts with PtdIns4P. PI3P is a unique modified lipid generated on endosomal membranes by monovalent PI3 kinase type III/VPS34, the only PI3K isoform expressed in *T*. *brucei* [25]. To test whether VPS34 was also involved in mediating ATG24 recruitment from the cytosol onto membranes in *T*. *brucei*, we incubated PCF wild-type cells with the PI3 kinase inhibitor wortmannin (0.3–30 μM), and followed the effect on the subcellular localization of ATG24 by immunofluorescence (Fig 3A). Treated cells showed a concentration-dependent release of ATG24 from puncta, resulting into more diffuse cytosolic labeling at 3 μM and higher concentrations, indicating that membrane association required PI3 kinase activity. The association of ATG24 to puncta dropped from 62% to 33% in the first experiment and from 42% to 25% in the second one, when comparing cells in normal conditions *versus* cells exposed for 1 h to 3 μM wortmannin. However, the specificity of this pharmacological inhibition could be questioned, since wortmannin is a broad-spectrum irreversible inhibitor of PI3 kinases. We therefore turned to specific silencing of VPS34 using a BSF RNAi cell line as an alternative approach. This RNAi cell line is considerably (70–80%) retarded in growth, over a 12-day period, from about 24 h after induction of decreased VPS34 expression (M. Herman and PM, unpublished result). Indeed, the importance of VPS34 for BSF *T*. *brucei* has been demonstrated previously [25]. In these cells, 24 h after VPS34 RNAi induction, ATG24 showed loss of immunolabeled puncta and a more diffuse distribution, confirming that association of ATG24 to endocytic vesicles requires VPS34 (Fig 3B).

### ATG24 is necessary for receptor-mediated endocytosis and controls flagellar pocket homeostasy

**Fig 4:**
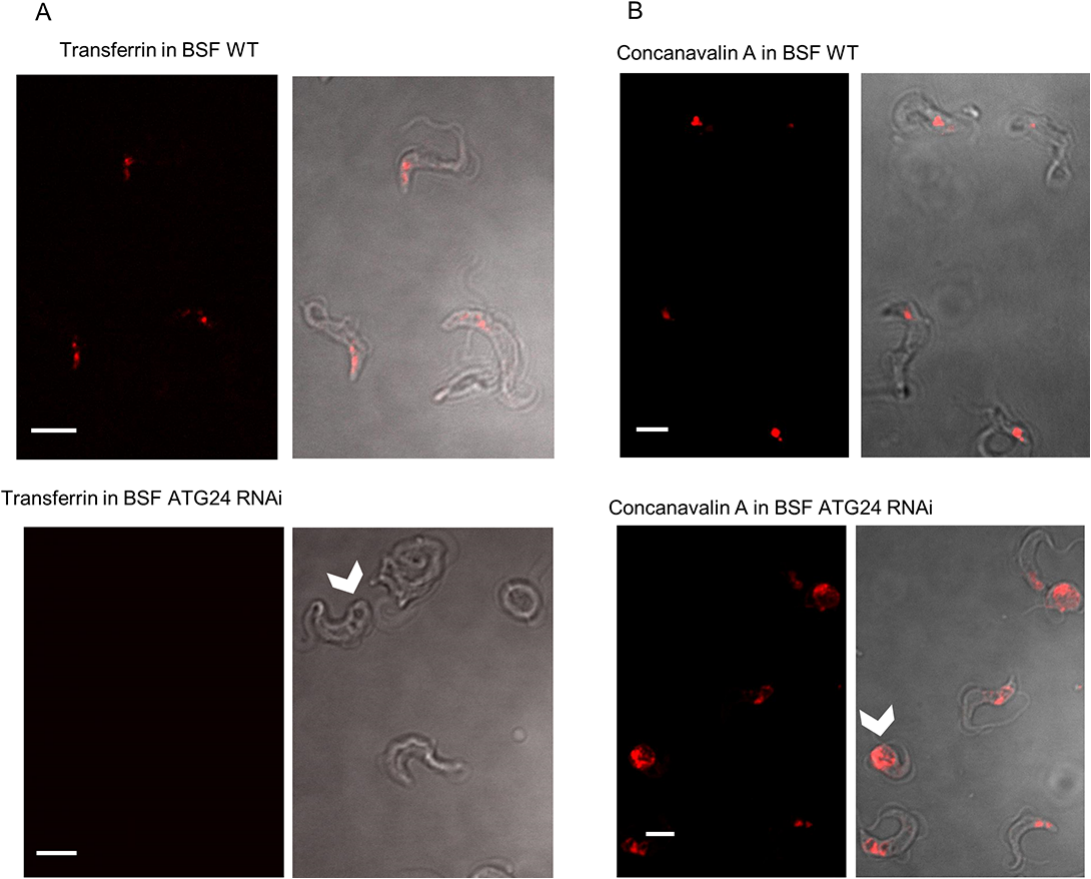
ATG24 silencing in *T*. *brucei* abrogates receptor-mediated endocytosis and induces enlargement of a single structure. Each pair combines fluorescence confocal imaging (left) with interference contrast (right). **A**) Fluorescence of Alexa-568 transferrin. BSF wild-type and ATG24 RNAi cells induced for 48 h were incubated at 37°C for 30 min with 6.25 nM Alexa568-transferrin. As illustrated here, most of the wild-type cells have internalized transferrin (red) into discrete structures, but the majority of ATG24 RNAi-expressing cells show no detectable fluorescence. The arrowhead indicates an enlarged structure not stained by transferrin. Quantification of transferrin labeling involved analyzing 24 wild-type and 107 ATG24 RNAi induced cells in the first experiment and 85 wild-type and 150 ATG24 RNAi cells in the second experiment. **B**) Fluorescence of concanavalin A. BSF wild-type and ATG24 RNAi-expressing cells (induced for 48 h) were incubated at 37°C for 20 min with 1.9 nM Alexa594-concanavalin A. Note the single concanavalin A-labeled structure (arrowhead). All images are from one representative experiment out of two. In the first experiment 42 wild-type and 61 ATG24 RNAi-induced cells were assessed for concanavalin A labeling, in the second experiment 25 wild-type and 206 ATG24 RNAi cells. All scale bars: 5 μm. The quantification method used is described in the Materials and Methods section.

We concluded that *T*. *brucei* ATG24 identified here was the most likely counterpart of the Atg24 in *S*. *cerevisiae* which participates in endocytosis and autophagy, notably pexophagy. Since endocytic tracing by confocal microscopy presented above indicated VPS34-regulated association of *T*. *brucei* ATG24 with the endocytic system, we further tested for a function of ATG24 in receptor-mediated endocytosis by vital imaging of fluorescent transferrin uptake in control *versus* induced BSF ATG24 RNAi cells. A representative figure for ATG24 depletion during ATG24 RNAi in BSF cells is shown in the Supporting Information (S2A Fig); the ATG24 level in RNAi non-induced cells had dropped to 46% compared to WT cells and to 10% in 24 h-induced cells. ATG24 silencing virtually abrogated intracellular transferrin labeling (Fig 4A); in two independent experiments, intracellular transferrin was detectable in 75% and 92% of the wild-type cells, but only in 11% and 25% of 48 h-induced ATG24 RNAi cells, indicating that ATG24 is indeed a necessary component of the endocytic machinery in this parasite.

Whereas uptake of transferrin was affected in RNAi-induced ATG24 cells, no change in uptake of concanavalin A was observed. However, in some of the RNAi-induced cells we noticed an enlargement of a structure that could be labeled by concanavalin A (Fig 4B). Since Koumandou et al. [23] reported that decrease of the level of the protein identified by us as the ATG24 by RNAi resulted in an increase the size of the lysosome in BSF, we performed immunofluorescence for the lysosomal marker p67 in our BSF ATG24 RNAi cell line (not shown), but observed no significant size difference for p67-labeled lysosomes between BSF wild-type, ATG24 RNAi non-induced and ATG24 RNAi 72 h induced cells.

**Fig 5:**
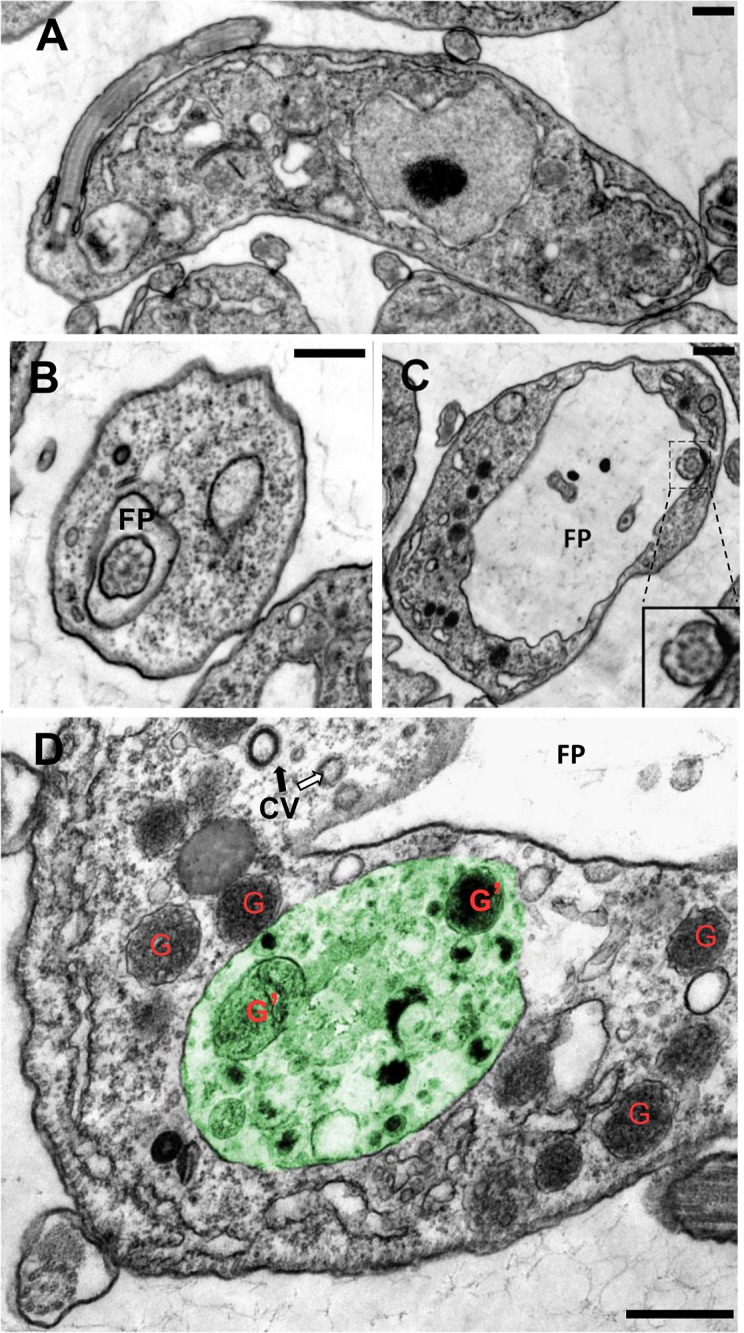
Ultrastructural alterations induced in *T*. *brucei* BSF by ATG24 silencing. Electron microscopy of BSF wild-type cells (**A, B**) and BSF ATG24 RNAi cells after 72 h of RNAi induction (**C**, **D**). The two characteristic effects of ATG24 silencing are (i) **enlargement of the flagellar pocket** (FP) shown in **C**, **D**; compare **B** with **C** (notice different scale bars); insert in **C** better shows a cross-section of the flagellum; and (ii) **autophagy** [**D**]. The large vesicle, painted in green, has a single limiting membrane and heterogeneous content including two well-recognizable entrapped glycosomes (G’), undistinguishable from free glycosomes (G) in the cytosol. This structure thus demonstrates active autophagy of glycosomes. The two arrows indicate CV (clathrin-coated vesicles): notice the contrast, thickness and indentation regularity typical of clathrin coat indicated by the black arrow, while the white arrow points to a suggestive additional CV profile. These structures suggest (some) preservation of endocytic entry. All scale bars: 0.5 μm.

Having ruled out the enlarged compartment labeled with concanavalin A in ATG24 RNAi cells as a swollen lysosome, we turned to electron microscopy which unambiguously identified enlarged structures as swollen flagellar pockets (Fig 5; compare panels B and C). An up to four-fold increase in sectioned diameter could be observed in some cells, indicating a >50-fold volume increase. However, we noticed considerable heterogeneity in the extent of flagellar pocket swelling, likely attributed to differences in ATG24 levels between individual RNAi cells. Partial knockdown with heterogeneity in the resulting phenotypic effects in individual trypanosomes is a well-known phenomenon. Considering that concanavalin A labeling was restricted to the flagellar pocket in cells where this structure was enlarged, we also conclude that these cells suffer an important block of bulk endocytosis. Note also that autophagy is seen in panel D, where cellular components including two glycosomes are observed surrounded by a membrane in this single section.

**Fig 6:**
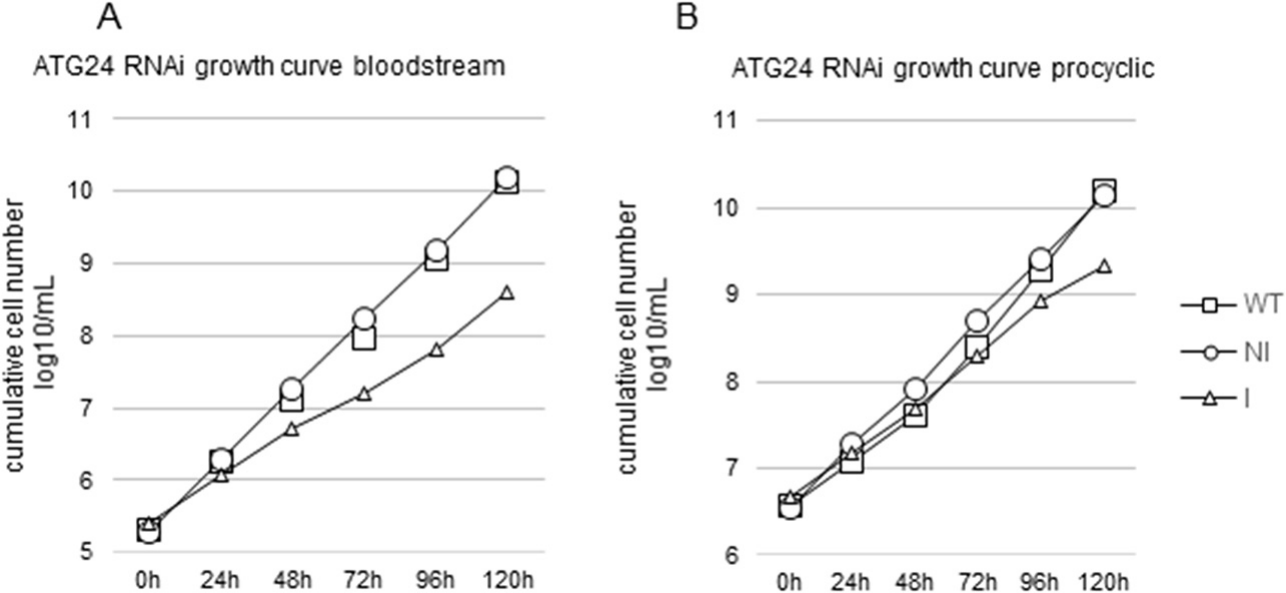
Growth curve for ATG24 RNAi cell lines. Minor growth inhibition by ATG24 silencing in *T*. *brucei* BSF (**A**) and PCF (**B**). Comparison of WT *vs* non-induced (NI) or induced (I) ATG24 RNAi cell line. WT–squares, NI–circles, I–triangles. Results are from one representative experiment out of three, giving the following population doubling times (PDT; mean ± SD): bloodstream-form wild-type cells 7.45 ± 0.61 h; ATG24 RNAi NI: 7.66 ± 0.48 h; ATG24 RNAi Ind: 11.51 ± 2.0 h. At 120 h the differences in PDT between BSF WT and ATG24 Ind and between ATG24 NI and ATG24 Ind are statistically significant with p-values of 0.012 in both cases, whereas the value for WT *versus* ATG24 NI is 0.708 and the difference thus not statistically significant. The PDT for procyclic trypanosomes are: WT, 9.16 ± 1.74 h; ATG24 RNAi NI, 10.17 ± 1.62 h; ATG24 RNAi Ind, 13.65 ± 3.6 h. At 120 h the p-value for differences in PDT of PCF WT *versus* ATG24 Ind is 0.204, ATG24 NI *versus* ATG24 Ind is 0.315, and WT *versus* ATG24 NI is 0.201; none of these differences is statistically significant.

Since receptor-mediated endocytosis is the only significant route for supply of essential nutrients such as iron and cholesterol in BSF trypanosomes [26,27], we next looked at cell growth. Reproducibly, upon ATG24 RNAi, growth of BSF cells was inhibited, noticeable two days after induction, but this effect was lower for PCFs (Fig 6, note the logarithmic scale). The two-day delay could be due to the time required for (partial) silencing and/or may be explained by a possible exhaustion of iron and/or cholesterol stocks in BSF. For PCF, the marginal growth inhibition could reflect incomplete depletion of the protein by RNAi and the less important role of endocytosis in this stage of the parasite’s life cycle than in the BSF.

### ATG8.1 and ATG8.2 as markers of autophagy in *T*. *brucei*

To next address the key question as to whether ATG24 silencing impacted on autophagy, we first evaluated ATG8.1 and ATG8.2 isoforms as autophagosome markers in *T*. *brucei*. Atg8/LC3, a classical autophagosomal marker protein, is conserved in all organisms where autophagy takes place. Atg8/LC3 function normally requires the proteolytic activity of Atg4/ATG4 to expose a C-terminal glycine residue that binds to phosphatidylethanolamine (PE) at the autophagosomal membrane. *T*. *brucei* possesses three different homologs of the single ATG8 yeast gene, named ATG8.1 (Tb927.7.5900 also known as ATG8A in TriTrypDB), ATG8.2 (Tb927.7.5910 or ATG8B) and ATG8.3 (Tb927.7.3320 or ATG12). The amino-acid sequences of the first two proteins are 80% identical, differing mainly in their N-terminal region, whereas the third is very different. Importantly, ATG8.1 exposes its C-terminal glycine and apparently does not require the proteolytic activity of ATG4 for interaction with the developing autophagosomal membrane. There is no report of a detrimental effect of overexpression of Atg8/LC3 proteins, however a recent paper surprisingly reported that *T*. *brucei* lacking ATG8.1 or ATG8.2 survive better when exposed to starvation conditions [28]. *T*. *brucei* ATG8 has been shown to participate in autophagy and to be a suitable marker for following the process [28–30].

**Fig 7:**
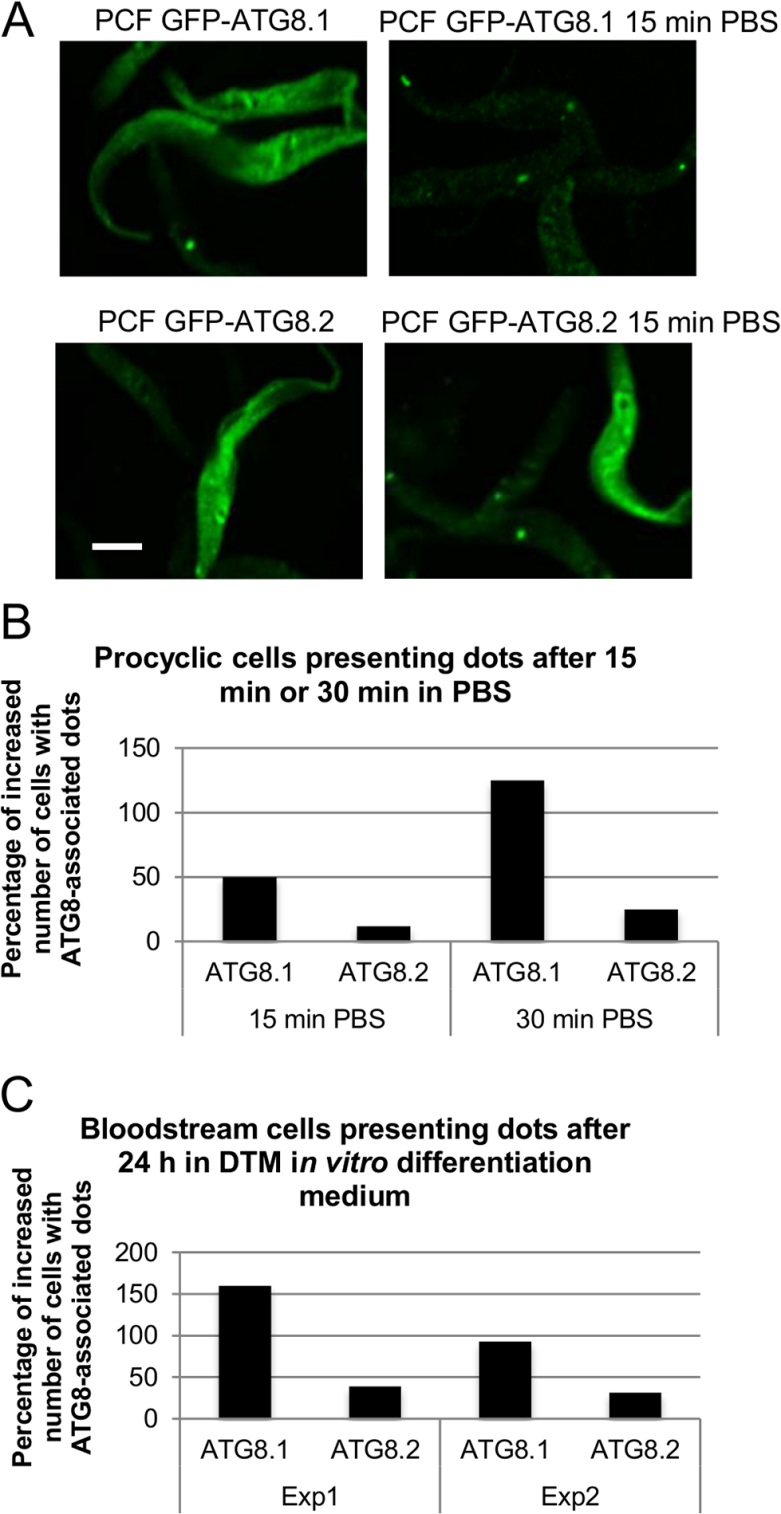
ATG24 depletion in *T*. *brucei* results in an increased number of ATG8-associated puncta. **A**) Validation of autophagosome labeling in *T*. *brucei*. Starvation-induced dotty labeling with GFP-ATG8.1 or GFP-ATG8.2 upon transfer of transfected PCF cells from control medium (left) to PBS for 15 min (right). All scale bars: 5 μm. **B**) Quantification of autophagy upon ATG24 silencing in PCF trypanosomes. Comparison of the percentage of increase in number of cells displaying puncta labeled for GFP-ATG8.1 or GFP-ATG8.2 before and after transfer to PBS of non-induced (NI) and induced (I) cells in two separate experiments (Exp1 with 15 min incubation in PBS and Exp2 with 30 min incubation in PBS); 50 to 200 cells were counted in each condition. All cells were fixed in the same manner in order to minimize different effects caused by fixation. Selected were only cells that had retained their structure and in which there was visible fluorescence. The fluorescence distribution in whole cells was analyzed and cells displaying fluorescent puncta versus cells in which GFP-ATG8 had only a cytosolic distribution were counted. **C**) Quantification of autophagy upon ATG24 silencing in BSF cells. Comparison of the percentage of increase in number of cells displaying puncta associated to GFP-ATG8.1 or GFP-ATG8.2 before and after 24 h in DTM *in vitro* differentiation medium in wild-type (WT), non-induced (NI) and induced (Ind) cells in two separate experiments (Exp1 and Exp2); at least 100 cells were counted in each condition. After harvesting, the cells were treated and analyzed as in the experiments shown in panel B.

To study autophagy in the parasite, we used two procyclic cell lines in which genome-inserted GFP-tagged ATG8.1 or ATG8.2 were constitutively expressed under the control of a PCF-specific procyclin promoter (constructs kindly provided by R. Schmidt and Dr P. Bütikofer, Bern, Switzerland). BSF cells possessing this construct were able to express the GFP-tagged proteins when placed in differentiation medium (not shown). In the PCF, autophagy was quantified by counting cells displaying fluorescent puncta, as an indication of GFP-ATG8 association with autophagosomes. Both transfected PCF cell lines showed dotty fluorescence as early as 15 min after transfer into PBS to induce starvation, confirming that both isoforms can participate in autophagosome formation in *T*. *brucei* and are valid autophagosomal markers in this organism (Fig 7A). Moreover, the levels of both ATG8 isoforms decreased upon prolonged incubation in PBS, as would be expected to result from lysosomal degradation in autophagy-inducing conditions (S2B Fig).

### ATG24 is a negative regulator of autophagy and *in vitro* differentiation

To assess if TbATG24 has a role in autophagy, the effect of its silencing on the response to starvation was tested by measuring the percentage of cells displaying GFP-ATG8.1- or GFP-ATG8.2-labeled puncta. In two independent experiments performed by exposing cells to starvation conditions for 15 or 30 min, this percentage was increased upon ATG24 silencing in PCF expressing GFP-ATG8.1, but not detectably for GFP-ATG8.2 (Fig 7B). The observed increase in number of cells displaying puncta in starvation conditions in the absence of ATG24 was approximately 50% after 15 min of starvation. When the cells were exposed for 30 min to starvation, the increase in the number of cells displaying puncta could reach 100%. Efficient differentiation of BSF cells is expected to trigger expression of the procyclin promoter-controlled GFP-ATG8.1 and GFP-ATG8.2. We thus scored the number of cells expressing GFP-ATG8.1 or GFP-ATG8.2, 24 h after transferring BSF cells to DTM, a medium allowing trypanosomes to differentiate. The absence of ATG24 was associated with a higher number of cells presenting puncta (Fig 7C).

The increase in ATG8 puncta may either indicate an increase of entry in autophagy or a block in the delivery of autophagosomes to the lysosome. It has been shown that transferring PCF cells from culture medium to PBS causes an increase in lysosomal size and colocalization of glycosomal proteins with the lysosomal marker p67 [15]. It is possible that the increase in GFP-ATG8 puncta caused by ATG24 depletion is not directly related to a role in autophagy, but a secondary effect of its role in endocytosis. Endocytosis in ATG24 depleted PCF cells was not investigated. However, the endocytosis rate in the PCF is much slower than in the BSF, where it is important not only for nutrient uptake but also for a very rapid (every 12 min) turnover of the entire surface coat to clear it from attached antibodies [31,32]. In view of the fast and important effect of ATG24 depletion on autophagy induction in PCF where endocytosis is not as relevant as it is for BSF, we consider therefore most likely that the observed effects are caused by a role of ATG24 in autophagy.

**Fig 8:**
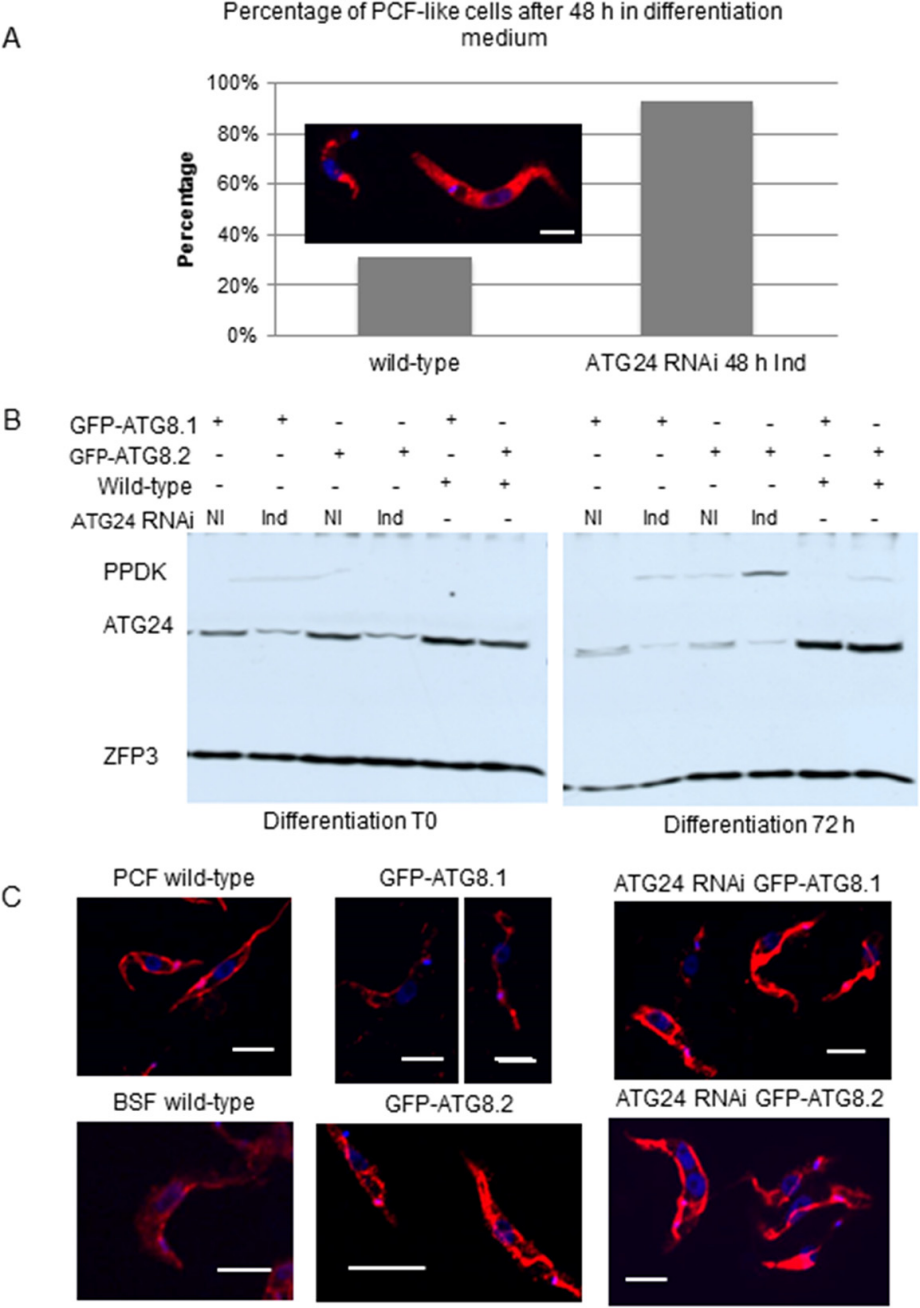
ATG24 silencing accelerates *T*. *brucei* differentiation. **A**) Morphological evidence. Immunofluorescence image at the upper left compares the typical appearance of a BSF (at left) and a PCF trypanosome (at right) labeled with antiserum against phosphoglycerate kinase (PGK). Quantification of differentiation for wild-type (WT) cells and ATG24 RNAi trypanosomes first induced for 48 h, then transferred to *in vitro* differentiation medium for 48 h. PCF-like morphology is seen in 31% ± 14% of the wild-type cells, but 93% ± 9% of ATG24 RNAi cells. 50 to 400 cells were counted for each sample from three independent experiments (p value = 0.002). **B**) PPDK expression was assayed by western blot analysis after 72 h in differentiation medium for wild-type and ATG24 RNAi non-induced (NI) or induced (Ind) trypanosomes expressing GFP-ATG8.1 or GFP-ATG8.2 as indicated. Partial depletion of ATG24 can be observed in these cells. ZFP3 is a loading marker, i.e. a protein known to remain rather constant during differentiation. **C**) An illustration of mitochondrial morphology, as revealed by ASCT staining, in wild-type BSF and PCF cells (as controls), BSF cells expressing GFP-ATG8.1 or GFP-ATG8.2 with and without ATG24 RNAi 48 h induced before *in vitro* differentiation (72 h). ASCT (red), DNA (blue, DAPI). Note the developed mitochondrion of the PCF cells in contrast to the BSF trypanosomes. After 72 h in differentiation conditions, the cells expressing GFP-ATG8.2 have a more developed mitochondrion than cells expressing GFP-ATG8.1, while in cells depleted for ATG24 there is no significant difference between those expressing tagged ATG8.1 and ATG8.2. All scale bars: 0.5 μm.

Since ATG24 silencing favored autophagy, we finally looked whether this was linked with a promotion of differentiation, using morphological and biochemical criteria. As shown by morphological scoring in Fig 8A, ATG24 silencing promoted by approximately 3-fold the conversion of LS into PCF-like as compared to wild-type cells under *in vitro* differentiation conditions (as seen also in Fig 7C). We also followed differentiation in cells overexpressing GFP-ATG8.1 or GFP-ATG8.2, using constructs in which expression of the tagged protein is controlled by a PCF-specific promoter. After 24 h in differentiation medium the BSF cells indeed expressed GFP-tagged ATG8.1 and GFP-ATG8.2 (western blots for GFP-ATG8.2 are shown in S2C Fig). Quite interestingly, ATG8.1 or ATG8.2 overexpression instead considerably *slowed down* differentiation in the absence of ATG24 silencing. This indicated that ATG8 overexpression by itself strongly impaired differentiation, but also that this effect could be overcome by ATG24 RNAi induction, providing additional evidence for a link between autophagy and differentiation. As a biochemical criterion, we used the induction of pyruvate, phosphate dikinase (PPDK; a glycosomal enzyme also known by its systematic name ATP:pyruvate, phosphate phosphotransferase) (Fig 8B), since this enzyme is known to be expressed in PCF but not in BSF cells [33]. This seems to confirm that absence of ATG24 increases the rate of differentiation (as seen by the presence of the PCF marker PPDK). Moreover, cells expressing GFP-ATG8.2 appeared to differentiate faster than cells expressing GFP-ATG8.1, as was evidenced by a higher level of PPDK (Fig 8B). This conclusion was corroborated by the observation of a more developed mitochondrion, by immunofluorescence, using acetate:succinate CoA-transferase (ASCT) as a marker, as observed after 72 h incubation in differentiation medium (Fig 8C). Mitochondrial development is a characteristic of PCF cells. Together, the data in Fig 8 support the notion that differentiation of trypanosomes expressing GFP-ATG8.2, after 72 h in differentiation medium, is more efficient than that of trypanosomes expressing GFP-ATG8.1.

The larger number of autophagosomes detected in PCF trypanosomes of the GFP-ATG8.1 cell line compared to the GFP-ATG8.2 cell line upon starvation (see above) might seem contradictory with the observation of a more efficient differentiation of the BSF cell line expressing GFP-ATG8.2, inferring that this efficient differentiation is the result of a higher autophagy rate. Two explanations can be postulated. First, it is possible that the two isoforms of ATG8 act differently in general autophagy and in the possibly controlled type of autophagy that takes place in *T*. *brucei* during its differentiation. Similar divergent roles have been observed for the distinct ATG8 families in *Leishmania major* [34]. Alternatively, ATG8.2 may indeed be more efficient than ATG8.1 in participating in autophagy causing an earlier appearance of a peak in the number of autophagosomes to which this protein is associated than in the cells expressing AGT8.1. At the time point at which cells were analyzed in our experiments, the higher number of autophagosomes labeled with ATG8.1 could reflect the delayed peak in the number of these autophagosomes. Herman et al. [15] reported an increase in the colocalization of glycosomes and the lysosomal marker p67 as soon as 10 min after placing procyclic cells in PBS, indicating that autophagy is rapidly upregulated when these cells are starved.

Furthermore, when BSF cells were incubated in differentiation medium for 24 h, the percentage of cells undergoing autophagy was increased upon ATG24 RNAi induction for 48 h as compared to wild-type and non-induced cells (Fig 7C). This might be considered as a strong argument for a genetic link between autophagy and stage differentiation, as will be discussed below.

## Discussion

The life cycle of *T*. *brucei* involves several stages successively adapted to the mammalian bloodstream and the midgut and salivary glands of the tsetse fly. Adaptation of *T*. *brucei* from each stage to the next one involves well-defined morphological and metabolic changes [35]. Our previous identification of colocalization of glycosomes (labeled by aldolase) with a lysosome marker (p67) in all cells differentiating from SS BSF to PCF glycosomes indicates that the population of glycosomes are turned over during the parasite’s differentiation [15], an observation for which autophagy is an attractive explanation. To test this hypothesis at the molecular level, we selected ATG24, because its counterpart in yeast is required for induction of specific autophagy, including pexophagy [21,22].

After having performed most of our work on the role of the protein encoded by Tb927.9.13380, identified by us as the equivalent of yeast ATG24, we became aware of the paper by Koumandou et al. [23] who had characterized this protein as the trypanosomatid counterpart of Vps5/SNX1. To assess if our previous identification was correct, we created evolutionary trees, using three different methods, with Tb927.9.13380 and PX domain-containing proteins from various organisms. This analysis suggested that Tb927.9.13380 is more closely related to Atg24/SNX4 than to Vps5/SNX1. The concordance in topology between trees calculated using different algorithms supports this conclusion despite the generally low bootstrapping values found.

We found that *T*. *brucei* ATG24 is expressed in both LS-BSF and in PCF cells, with slightly higher expression levels in the former. ATG24 apparently distributes in two pools, the cytosol and puncta, to which they are recruited in a PI3K/VPS34-dependent process, suppressed by wortmannin or upon VPS34 silencing, as is the case for yeast Atg24 and their mammalian homolog sorting nexin 4 (SNX4) [22,36]. Some of these puncta were identified as endocytic vesicles, based on their prevalent location between the nucleus and kinetoplast and vital labeling by internalized transferrin [37].

In our experiments, ATG24 silencing in *T*. *brucei* did not severely affect cell growth of either BSF or PCF cells. This observation contrasts markedly with those of Koumandou et al. [23] who reported the protein to be essential for *T*. *brucei*, based on BSF death upon ATG24 RNAi. In our experiments, ATG24 RNAi was compatible with survival and even growth although at a slower pace, likely due to partial silencing in our experiments (e.g. see S2A Fig and Fig 8B). The different extent of knockdown could be caused by the different vectors used, the hairpin-RNA encoding pHD677-based construct and a p2T7-based one using opposing promoters, in the work by us and Koumandou et al., respectively. Nonetheless, long-slender BSF cells partially depleted for ATG24 showed clear morphological defects, such as flagellar pocket enlargement in about one third of the cells, and functional impairment of receptor-mediated endocytosis of transferrin. In contrast, the uptake of concanavalin A was not qualitatively affected in most of our RNAi cells, raising the possibility of a specific effect on transferrin receptor recycling. This is in agreement with the key role of the ATG24 ortholog in mammalian cells, known as SNX4, in transferrin receptor recycling [24,38]. Importantly, transferrin import into cells is not associated with retromer components [39–42], another indication that the protein described by us as *T*. *brucei* ATG24 is more closely related to Atg24/SNX4 than to Vps5/SNX1.

Conceivably, the functions of Tb927.9.13380 encompass both those that are Atg24/SNX4-like, as indicated by its observed role in transferrin import and autophagy (see also below) and those elsewhere carried out by Vps5/SNX1 as observed by Koumandou and colleagues [23]. Possibly, the ancestral role of the protein was in endocytosis. ATG24 then acquired an additional, very different role in autophagy in yeasts and trypanosomatids (see below). One could further speculate that either Vps5/SNX1 was secondarily lost from the trypanosome, with Atg24/SNX4 taking over its function, or that trypanosomatids never possessed it. Based on the phylogenetic analysis and the taxonomic data of Koumandou et al. [23], we cautiously favor the former explanation. The function of the second SNX-like protein detected by our search and indicated in the phylogenetic tree (Tb927.7.4500) remains unknown.

Besides phylogenetic analysis, the notion that Tb927.9.13380 functions as an ATG24 is strongly supported by its involvement in autophagy. Both BSF and PCF trypanosome populations with knocked-down protein expression exhibited a higher proportion of cells presenting autophagosomes than wild-type and non-induced cells, and under two different autophagy-inducing conditions: transfer of PCF cells from rich medium into PBS and *in vitro* differentiation of BSF cells. In yeast cells, Atg24 interacts with components of the Atg1 autophagy-inducing complex and is required for several specific types of autophagy including pexophagy, mitophagy and cytoplasm-to-vacuole targeting of vacuolar enzymes, while its absence has no effect on general autophagy. Our data clearly show that *T*. *brucei* ATG24 has a role in general autophagy, quite different from that of its ortholog in yeast. In *P*. *pastoris* and *S*. *cerevisiae*, Atg24 is required for pexophagy. We found that depletion of TbATG24 affects autophagy during general starvation. However, the ATG24 RNAi-induced population had more cells undergoing autophagy than non-induced cells. This could indicate that in *T*. *brucei* ATG24 exerts a suppressor role on autophagy, since autophagy is enhanced when this negative effector is depleted.

It has been reported that both endocytosis and autophagy levels increase during differentiation of *T*. *brucei*, the former process even to surprisingly high levels in the SS stage [43]. Although our *in vitro* assay did not specifically address this stage, it provided also indications for increased autophagy during differentiation. Moreover, our work supports the notion that ATG24 is involved in both autophagy and endocytosis, but with opposing effects; endocytosis is reduced in cells partially depleted for the protein, whereas autophagy is increased and differentiation takes place faster than in wild-type cells.

Our observations on *T*. *brucei* ATG24 are also remarkably similar to those recently reported for TBC1D14 in mammalian cells, where it is located on recycling endosomes and involved in transferrin uptake, but acts as a negative regulator of starvation-induced autophagy. Interestingly, upon amino acid starvation, TBC1D14 relocalizes to the Golgi complex [44]. In BSF *T*. *brucei*, membrane recycling in the flagellar pocket takes place at an extremely high rate [26], which likely explains why imbalanced, but not arrested endocytic trafficking could result in such dramatic pocket enlargement. The functional consequences for the parasite deserve to be studied in more detail.

Of note, a recent high-throughput assay identified that, along with other components of the endocytic system and lysosomal proteins, ATG24 (Tb927.9.13380) is involved in facilitating the uptake of suramin, the recommended drug for the treatment of first-stage sleeping sickness caused by *T*. *b*. *rhodesiense* [45]. The drug cannot passively cross the membrane due to its large size and high negative charge, and its uptake and delivery to the lysosome seem to involve ATG24 as well as the surface protein ISG75.

The strong acceleration of BSF differentiation upon ATG24 silencing is a major result of our study. A linkage with promotion of autophagy is tempting, although independent effects cannot be excluded. In contrast, overexpression of GFP-ATG8.1 or GFP-ATG8.2 under the strong constitutive procyclin promoter slowed down differentiation in the absence of ATG24 silencing. These observations indicate that excess ATG8 in *T*. *brucei* may be deleterious, either because autophagy becomes uncontrolled so as to cause excessive degradation of important cytosolic material or organelles, or due to a dominant-negative effect. However, the correct localization to puncta and the widespread use of GFP-tagged ATG8 indicate that the first explanation is more likely. Our observations appear to be in good agreement with a recent study showing that silencing either ATG8.1 and/or ATG8.2 in trypanosomes increased resistance to starvation [28]. However, reversion of growth inhibition upon ATG24 silencing argues against a mere toxic effect and rather points to interaction with the autophagic machinery. Unfortunately, we were not able to measure the autophagy rate in wild-type cells without the tag and therefore could not determine if the cells expressing the tagged ATG8 had a higher or lower autophagy rate when compared to wild-type trypanosomes. The reason why the GFP tag-expressing cells are less successful in differentiating remains unclear.

The different effects of the two ATG8 isoforms on autophagy and the differentiation rate deserve some comment. Since both are expressed from the same genomically-inserted vector, similar expression levels should be expected; yet we observed a lower level of ATG8.1 than ATG8.2. The difference is more likely related to a different control by ATG4 peptidase before interaction with the forming phagophore membrane. Indeed, the C-terminal cysteine residue of ATG8.2 likely needs processing by the peptidase activity of ATG4 in contrast to ATG8.1 that has its C-terminal glycine residue already exposed.

Taken together we conclude that autophagy and differentiation are closely linked in *T*. *brucei* and that ATG24 acts as a repressor of autophagy and has an effect on ATG8, thereby controlling the induction or intensity of the process.

## Materials and Methods

### Trypanosomes and growth conditions

For all experiments, we used monomorphic BSF and PCF cells of *T*. *brucei* Lister 427, cell line 449 (gift from Prof. C. Clayton, Heidelberg) [46]. These parasites constitutively express the *Escherichia coli* tetracycline (Tet) repressor gene from a chromosomally integrated plasmid pHD449, that also endows phleomycin resistance. This cell line is metabolically indistinguishable from the wild type. BSF stage parasites were maintained in HMI-9 medium supplemented with 10% heat-inactivated fetal calf serum (Invitrogen, Gibco-10437-028) as well as 0.18 μg/mL phleomycin (Cayla, ant-ph) and incubated at 37°C under water-saturated air with 5% CO_2_. PCF cells were cultured in SDM79 medium containing 15% fetal calf serum and 0.5 μg/mL phleomycin, at 28°C under water-saturated air with 5% CO_2_. Experiments were performed with cells in the exponential phase of growth, *i*.*e*. < 2x10^6^ cells/mL for the BSF and <5x10^7^ cells/mL for the PCF.

A VPS34 RNAi derivative of the BSF *T*. *brucei* 449 cell line [46] has been prepared previously in our laboratory (M. Herman and PM, unpublished data). This cell line, harboring the genomically integrated vector pHD1336 containing a fragment of the *VPS34* gene, has shown a severely reduced growth rate upon VPS34 depletion. In the study reported here, the cell line was cultured in the presence of 5 μg/mL blasticidin (Invitrogen, R21001) to maintain selection pressure. VPS34 RNAi was induced for 24 h before samples were collected for immunofluorescence.

### Identification of the sequence for *T*. *brucei* ATG24, phylogenetic analysis

The identification of the sequence for the trypanosomatids putative homologs of several yeast ATGs including ATG24 has been described by Herman et al. [14]. The gene (TriTryp database: http://tritrypdb.org/tritrypdb/, accession code: Tb927.9.13380, formerly Tb09.211.4240) codes for a protein of approximately 42.8 kDa that contains a Phox (PX) homology domain in its N-terminal region and a BAR (Bin/Amphiphysin/Rvs) in its C-terminal domain. It is 24% identical to its yeast counterpart.

To determine evolutionary relationships between the candidate TbATG24 and other proteins comprising both a PX and BAR domain in *T*. *brucei* and some other organisms (*S*. *cerevisiae*, human, *Drosophila melanogaster* and *Caenorhabditis elegans*), their sequences were first aligned for optimal positional identity. Terminal regions upstream of the PX domain and downstream of the BAR domain which were clearly non-homologous were trimmed. The relationships between the truncated sequences were then analyzed by three different methods: the Neighbor-Joining (NJ) method [47], the Minimum Evolution (ME) method [48] and the Maximum Parsimony (MP) method [49]. The analysis involved 27 amino-acid sequences. There were a total of 629 positions in the final dataset. Evolutionary analyses were conducted in MEGA5 [50]. In all three cases, a bootstrap consensus tree was inferred from 500 replicates. When the evolutionary history was inferred using the NJ method, an optimal tree with the sum of branch length = 17.12554452 was prepared. The evolutionary distances were computed using the p-distance method [51] and are expressed in the units of the number of base differences per site. The ME tree was searched using the Close-Neighbor-Interchange (CNI) algorithm [51] at a search level of 1. The NJ algorithm was used to generate the initial tree. In the MP analysis, the most parsimonious tree with length = 5185 was used. The consistency index is (0.638254), the retention index is (0.449044), and the composite index is 0.296880 (0.286604) for all sites and parsimony-informative sites (in parentheses). The MP tree was obtained using the Close-Neighbor-Interchange algorithm [51] with search level 1 in which the initial trees were obtained with the random addition of sequences (10 replicates).

### Protein expression and antiserum production

The complete *ATG24* gene sequence was amplified by PCR using Phusion polymerase (New England BioLabs Inc., M0530S) and the following pair of primers: 5’-CCACAT*ATG*TCTGAAAATGTCTTTGAGTTCCGT-3’ (forward, containing a *Nde*I restriction site, shown underlined and the start codon of the gene italicized) and 5’-CCATCTCGAG *CTA*ATCGTCATCAATCAAATG-3’ (reverse, containing a *Xho*I restriction site, shown underlined and the stop codon of the gene italicized). The amplified product was purified and ligated into the expression vector pET28a (Novagen, 69864–3), that allows the expression of the protein with a N-terminal (His)_6_-tag. Purified recombinant protein was used for rabbit immunization to raise a polyclonal antiserum (Gentaur).

### Preparation of DNA constructs

A construct for the expression of ATG24 with at its C-terminus fused to GFP was made by PCR, using the following primers: (forward) 5’-CCCAAGCTT *ATG*TCTGAAAATGTCTTTGAGTTC-3’ (*Hind*III site underlined and start codon italicized) and (reverse) 5’-AGGGGATCCATCGTCATCAATCAAATGGT-3’ (no stop codon, *Bam*HI site underlined) and GoTaq DNA polymerase (Promega, M3175). The amplified PCR product was inserted into pGN1, a vector that can be integrated into the *T*. *brucei* chromosome and codes for a tetracycline-inducible GFP that can be expressed as a tag fused to the C-terminus of the protein coded by the DNA insert [52]. BSF cells were transfected using the method described by McCulloch et al. [53] and transfected cells were selected by their resistance to 5 μg/mL hygromycin (Sigma-Aldrich, H7772-1G). Transfected cells were maintained under hygromycin selection.

The *T*. *brucei* ATG24 RNAi construct was made using the pHD677 vector. A region of the gene consisting of 486 bp and a 50 bp shorter and complementary region were amplified from the *T*. *brucei* genome using the primers: (forward sense) 5'-GTAAAGCTTCCTGCATGAATTTGGTGTTGC–3' (*Hind*III site underlined), (forward antisense) 5'–TTACTCGAGTCTGCAGGTCAGCAAACAT–3' (*Xho*I site underlined), (reverse sense) 5'–TTACTCGAGCACGATGGAAGCGATGAATTT–3' (*Xho*I site underlined), (reverse antisense) 5'–GTAGGATCCCCTGCATGAATTTGGTGTT-3' (*Bam*HI site underlined). Upon transfection, the *Not*I linearized pHD677 vector will become inserted into a transcriptionally silent ribosomal RNA gene repeat spacer of the parasite's genome. As there are several positions where the plasmid can be inserted, and more than one plasmid can be inserted, the intensity of the RNAi phenotype may vary in different clones. Selection and maintenance of positive clones was done using hygromycin at 5 μg/mL for bloodstream-form cells and at 50 μg/mL for procyclics. Induction of RNAi was achieved by adding tetracycline at 1 μg/mL and 5 μg/mL for bloodstream- and procyclic-form trypanosomes, respectively. Experiments were performed using mid-log phase cultures.

For expression of GFP-tagged ATG8.1 and ATG8.2, BSF and PCF wild-type and ATG24-RNAi cells were transfected with EGFP-tagged TbATG8.1 and TbATG8.2 in the vector pG-EGFP-ΔLIIγ. These two plasmids were kindly provided by Remo Schmidt and Dr Peter Bütikofer (Bern, Switzerland). The linearized vector can integrate upstream of the tandemly-arranged procyclin genes and is controlled by a procyclin promoter. Consequently, the GFP-tagged proteins are constitutively expressed in PCF cells. Positive clones were selected by their resistance to G418 (Invitrogen, Gibco-11811-023) and maintained under G418 selection at a 15 μg/mL for BSF and 50 μg/mL for PCF. Although BSF cell lines expressed undetectable GFP levels by western blotting, dotty fluorescence could be observed by confocal microscopy.

### Confocal microscopy

BSF wild-type and ATG24 RNAi cells induced for 48 h were harvested while in the exponential phase of growth by centrifugation at 1,000 x *g* for 10 min at 4°C. The cells were washed in FCS-free HMI-9 with 1% BSA and resuspended in the same medium. Samples were incubated at 37°C for up to 30 min to assure the presence of ligand-free receptors, then allowed to take up Alexa568-transferrin or Alexa594-concanavalin A (each 5 μg/mL) (Life Technologies, T23365 and C11253, respectively) for up to 30 min, then placed on ice, washed in FCS-free HMI-9 with 1% BSA and processed for fluorescence microscopy.

After uptake, cells were washed in cold vPBS (Voorheis’ modified PBS, containing 46 mM sucrose and 10 mM glucose), and slides were mounted with DAKO (Agilent Technologies, S302380).

For immunofluorescence, trypanosome cultures were centrifuged at 2,200 x *g*, resuspended in PBS, fixed in 8% formaldehyde and permeabilized with 0.1% Triton X-100. After centrifugation and resuspension in PBS, cells were placed on poly-L-lysine treated slides. Blocking was performed with 5% BSA in water; antibodies were diluted in 2% BSA in water at the following dilutions. Rabbit anti-PGK-c (1:1,000), anti-ATG24 (1:400), anti-ASCT (gift from Dr F. Bringaud, Bordeaux; 1:750); mouse monoclonal anti-TPI (1:5,000); Alexa568-anti-rabbit (red; 1:500) and Alexa488-anti-mouse (green; 1:500) (both from Invitrogen) at 1:500. Hoechst 33342 was used at 0.2 μg/mL (324 μM). Slides were mounted with Mowiol 4–88 (Calbiochem, 475904-100GM) or DAKO fluorescent mounting medium and observed in a Zeiss Cell Observer Spinning Disc confocal microscope.

For p67 immunofluorescence, trypanosomes were centrifuged at 1,900 x *g*, resuspended in HMI-9, fixed in 2% formaldehyde and resuspended in PBS. The cells were then placed on silanized slides and permeabilized with 0.2% Nonidet P-40 in PBS. Blocking was done with 1% BSA in PBS and primary (monoclonal anti-p67, kindly provided by Prof. Michael Boshart, Ludwig Maximilians Universität, München) and secondary (Alexa594-anti-mouse, Molecular Probes, A-21205) antibodies were diluted 1:1000 in 1% BSA in PBS. 0.1 μg/mL DAPI was used at a 1:1000 dilution. Slides were mounted with VectaShield (Vector Labs, H-1000) and observed in a personal Delta Vision microscope. Images were analysed with the programs ImageJ or Fiji. When indicated, colocalisation between two markers was quantified as Pearson's correlation coefficient, calculated by the JACoP v2.0 Plugin of ImageJ [54].

Quantification of the ATG24-associated fluorescence intensity in a region selected because representing endosomes versus a region with only cytosol in the VPS34 RNAi experiment was done with the Fiji program to assign the mean intensity value for each selected area measured. For each cell a selection was made of an area representing the endosome as well as of a different area representing the cytosol and in each picture also an area representing the background was selected. The intensity value of the background was subtracted from both cytosol and endosome values and then the cytosol value was subtracted from the endosome value. The mean of the final endosome values were compared between WT and VPS34 RNAi 24-h induced cells.

### Western blotting

Protein samples were boiled for 5 min in Laemmli loading buffer, size-fractionated by SDS/PAGE and transferred to Amersham Hybond-ECL (GE Healthcare, RPN2020D) membranes. After blocking with 5% milk in PBS for at least 1 h at room temperature or overnight at 4°C under agitation, membranes were incubated with antibodies for 1 h at room temperature or overnight at 4°C under agitation in 1% milk in PBS. The primary antibodies, all raised in rabbits, were used at the following dilutions: ATG24 (1:4,000); RAB11 (1:2,000, gift from Prof. M. Field, Dundee); ZFP3 (1:10,000, gift from Prof. K. Matthews, Edinburgh); PPDK (gift from Dr F. Bringaud, Bordeaux; 1:25,000). As secondary antibody, peroxidase-conjugated goat anti-rabbit IgG; Invitrogen) was used at 1:10,000. After extensive washing in PBS then 0.05% NP40 in PBS, blots were revealed using Pierce ECL western blotting substrate (Thermo Scientific, 32106).

### *In vitro* differentiation

Cultures of BSF wild-type, ATG24 RNAi non-induced and 48 h induced trypanosomes, expressing or not the EGFP-ATG8.1 or EGFP-ATG8.2 constructs, were harvested in their exponential phase of growth at 37°C by centrifugation at 1,000 x *g* for 5 min. The cells were resuspended in pre-warmed (28°C) Differentiating Trypanosome Medium (DTM) [55] in the presence of 3 mM citrate and 3 mM cis-aconitate (from a 300 mM stock in 25 mM Hepes, pH 7.3), and kept at 28°C. Maintenance of clones was assured by culturing in presence of 1 μg/mL hygromycin and/or 15 μg/mL G418 when needed. RNAi induction was initiated by addition of 1 μg/mL tetracycline.

Differentiation was assayed by following the expression of the glycosomal procyclic PPDK, changes in cellular morphology in conjunction with changes in the subcellular localisation of PGK, which is mainly observed as a glycosomal protein (PGK-c) in BSF cells and as a cytosolic protein (PGK-b) in PCF cells.

### Autophagy-inducing assays

For general autophagy induction, PCF overexpressing GFP-ATG8.1 or GFP-ATG8.2 were centrifuged and resuspended in pre-warmed (28°C) PBS for up to 3 h. For initiating autophagy in BSF cells, *in vitro* differentiation was induced. When ATG24 depletion was required, autophagy and differentiation experiments were performed after 48 h of RNAi induction.

### Electron microscopy

Cells were collected by centrifugation at 1,000 x *g* for 10 min at 4°C, washed in PBS (pH 7.3), pelleted and centrifuged again, resuspended in PBS-Ca^2+^/Mg^2+^ (PBS, pH 7.0, 3.6 mM CaCl_2_, 3.0 mM MgCl_2_) and fixed by 2% glutaraldehyde in phosphate buffer at 4°C for 30 min. Cells were then transferred to veronal buffer and post-fixed with 1% osmium tetroxide in same buffer at 4°C for 1 h. Cells were washed again with veronal buffer, and pelleted in 2% agar. Minced pellet fragments were stained “en bloc” using 0.5% uranyl acetate in veronal buffer, overnight at 4°C and protected from light, then extensively washed, dehydrated in graded ethanol and embedded in Spurr. Ultrathin sections (70 nm) were obtained using a Reichert ultramicrotome and sections were further stained with uranyl acetate and lead citrate for 10 min each. Samples were observed in a transmission electron microscope (Philips, CM12) at 80 kV and images were imported to and analysed in AxioVision software (4.8.2; Zeiss, Germany).

## Supporting Information

S1 Fig Phylogenetic analysis to identify TbATG24. Bootstrapped Phylogenetic reconstructions using the Neighbor-Joining (**A**) and Maximum Parsimony methods (**B**) of the two PX-BAR-containing proteins from *T*. *brucei* (Tb; one of which Tb927.9.13380, is here referred to as TbATG24) and PX-BAR containing proteins from other organisms: Sc–*Saccharomyces cerevisiae*, Hs–*Homo sapiens*, Ce–*Caenorhabditis elegans*, Dm–*Drosophila melanogaster*. The numbers near the nodes indicate the percentage of replicate trees in which the associated taxa clustered together in the bootstrap test (500 replicates).
(TIFF)

S2 Fig Western blots of *Trypanosoma brucei*. (**A**) ATG24 expression and depletion in bloodstream (BSF WT, left panel) and procyclic wild-type *T*. *brucei* cells (PCF WT, right panel) and RNAi cells Non Induced (NI) and 48 h induced, as assayed with anti-TbATG24; cytosolic GAPDH (cGAPDH) was used as a loading control. The anti-ATG24 recognizes a single 48 kDa band in the lysates of the *T*. *brucei* cells. (**B**) GFP-ATG8.1 and GFP-ATG8.2 expression in procyclic *T*. *brucei* cells (PCF) in nutrient-rich medium (SDM79) and after 3 h of incubation in PBS to induce starvation, assayed with anti-GFP. ZFP3 (zinc finger protein 3) was used as a loading control. (**C**) GFP-ATG8.2 expression in two clones of bloodstream-form (BSF) trypanosomes in HMI9 medium and 24 h or 48 h after induction of their differentiation in DTM medium, as assayed with anti-GFP. Anti-PPDK was used as glycosomal marker, to assess the differentiation of BSF trypanosomes to ‘PCF-like’ cells.
(TIFF)
