# Supplementary figures and images for: ATG24 Represses Autophagy and Differentiation and Is Essential for Homeostasy of the Flagellar Pocket in Trypanosoma brucei
Source: PLoS One. 2015 Jun 19;10(6):e0130365. doi: 10.1371/journal.pone.0130365 (PMC4474607; doi:10.1371/journal.pone.0130365)

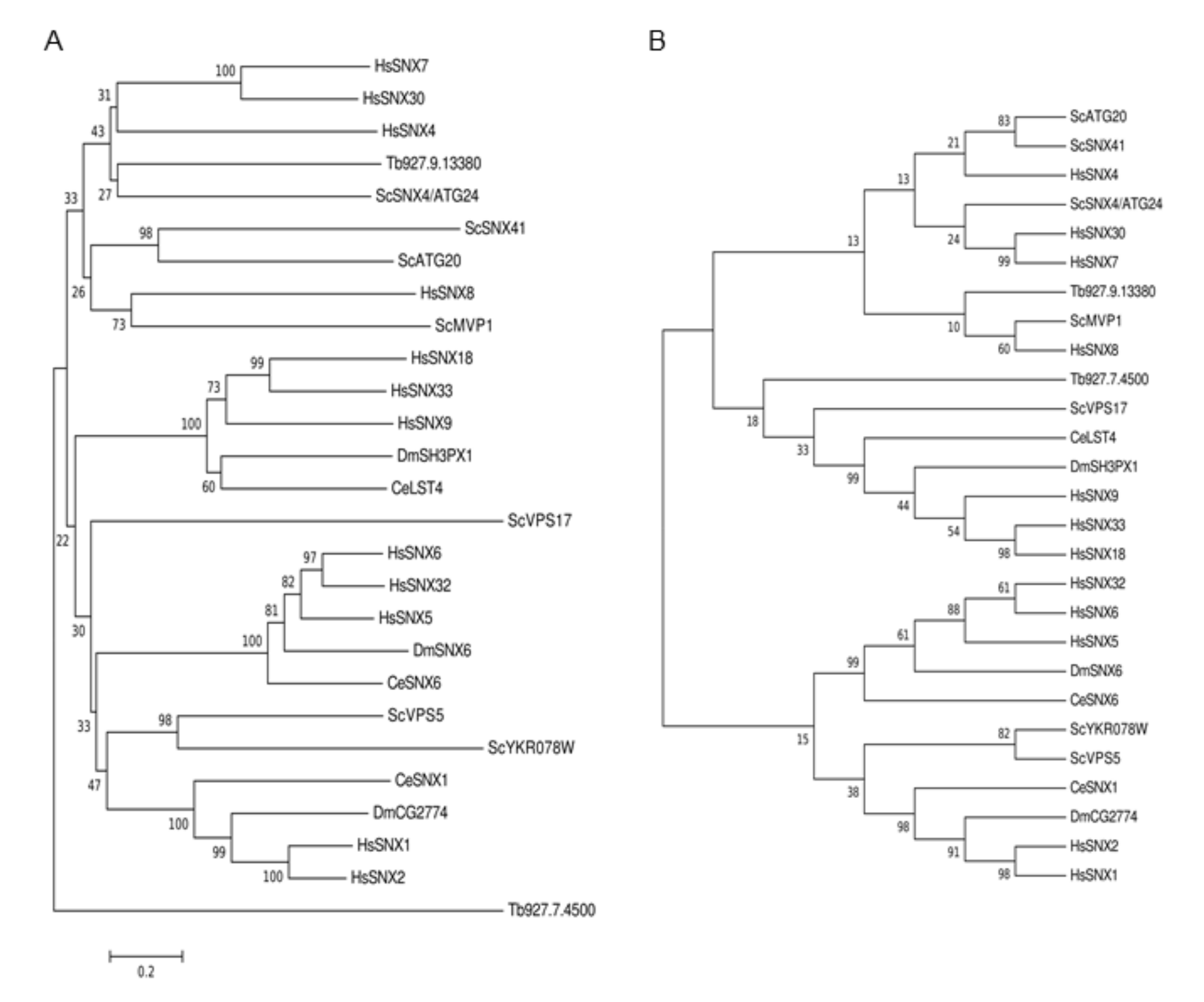

Supplement: S1 Fig — Bootstrapped Phylogenetic reconstructions using the Neighbor-Joining (A) and Maximum Parsimony methods (B) of the two PX-BAR-containing proteins from T. brucei (Tb; one of which Tb927.9.13380, is here referred to as TbATG24) and PX-BAR containing proteins from other organisms: Sc–Saccharomyces cerevisiae, Hs–Homo sapiens, Ce–Caenorhabditis elegans, Dm–Drosophila melanogaster. The numbers near the nodes indicate the percentage of replicate trees in which the associated taxa clustered together in the bootstrap test (500 replicates). (TIFF) [file pone.0130365.s001.tiff]

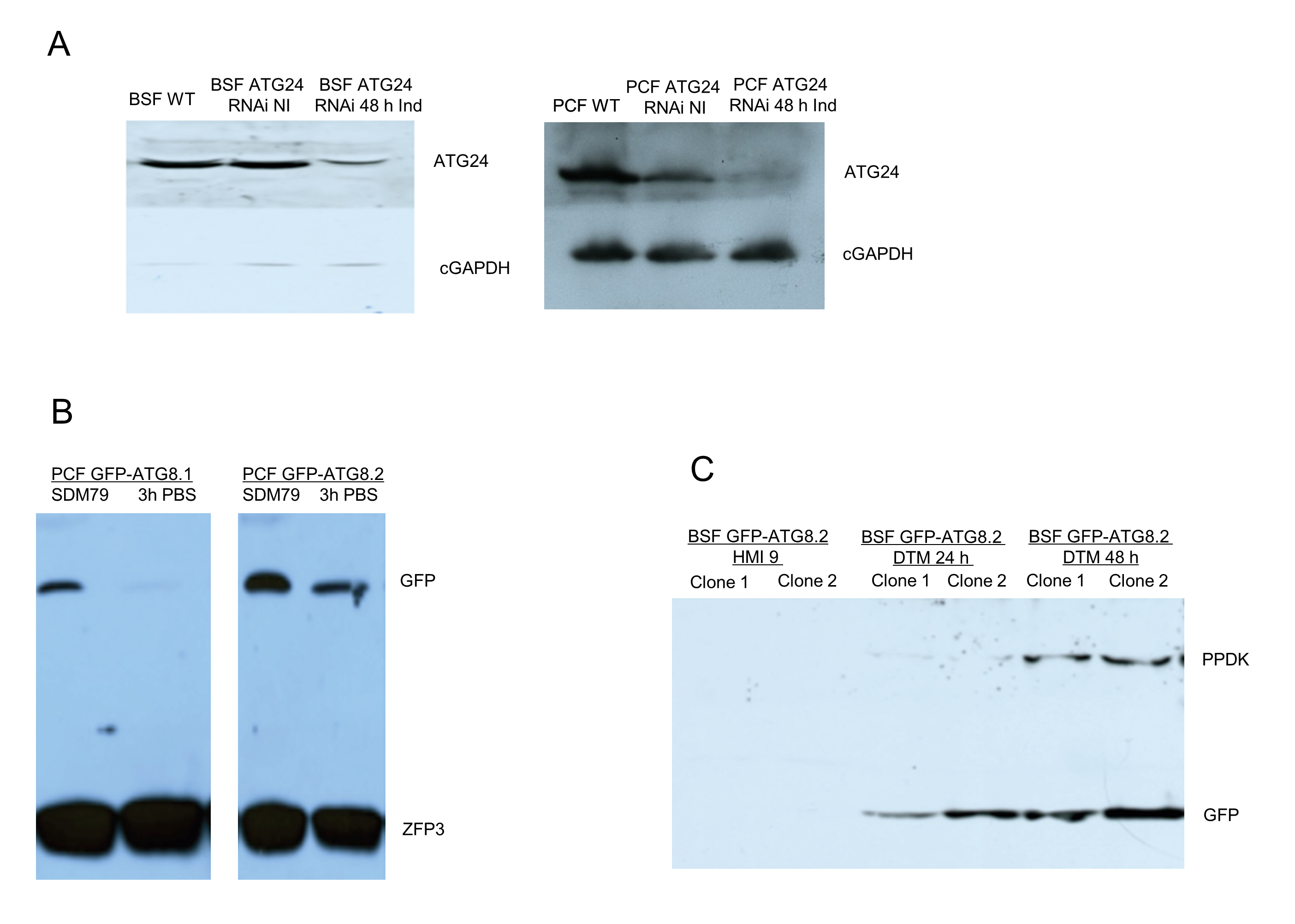

Supplement: S2 Fig — (A) ATG24 expression and depletion in bloodstream (BSF WT, left panel) and procyclic wild-type T. brucei cells (PCF WT, right panel) and RNAi cells Non Induced (NI) and 48 h induced, as assayed with anti-TbATG24; cytosolic GAPDH (cGAPDH) was used as a loading control. The anti-ATG24 recognizes a single 48 kDa band in the lysates of the T. brucei cells. (B) GFP-ATG8.1 and GFP-ATG8.2 expression in procyclic T. brucei cells (PCF) in nutrient-rich medium (SDM79) and after 3 h of incubation in PBS to induce starvation, assayed with anti-GFP. ZFP3 (zinc finger protein 3) was used as a loading control. (C) GFP-ATG8.2 expression in two clones of bloodstream-form (BSF) trypanosomes in HMI9 medium and 24 h or 48 h after induction of their differentiation in DTM medium, as assayed with anti-GFP. Anti-PPDK was used as glycosomal marker, to assess the differentiation of BSF trypanosomes to ‘PCF-like’ cells. (TIFF) [file pone.0130365.s002.tiff]
